# Supermagnetic Sugarcane Bagasse Hydrochar for Enhanced Osteoconduction in Human Adipose Tissue-Derived Mesenchymal Stem Cells

**DOI:** 10.3390/nano10091793

**Published:** 2020-09-09

**Authors:** Min Kim, Seung-Cheol Jee, Jung-Suk Sung, Avinash A. Kadam

**Affiliations:** 1 Department of Life Science, College of Life Science and Biotechnology, Dongguk University-Seoul, 32, Dongguk-ro, Ilsandong-gu, Goyang-si, Gyonggido 10326, Korea; pipikimmin@naver.com (M.K.); markjee@naver.com (S.-C.J.); sungjs@dongguk.edu (J.-S.S.); 2 Research Institute of Biotechnology & Medical Converged Science, Dongguk University-Seoul, 32, Dongguk-ro, Ilsandong-gu, Goyang-si, Gyonggido 10326, Korea

**Keywords:** sugarcane bagasse, hydrochar, hADMSCs, osteoconduction enhancement, hydrothermal carbonation

## Abstract

Hydrothermally carbonized sugarcane bagasse (SCB) has exceptional surface properties. Looking at the huge amount of SCB produced, its biocompatible nature, cheap-cost for carbonization, and its easy functionalization can give impeccable nano-biomaterials for tissue engineering applications. Herein, sugarcane bagasse was converted into hydrochar (SCB-H) by hydrothermal carbonation. The SCB-H produced was further modified with iron oxide (Fe_3_O_4_) nanoparticles (denoted as SCB-H@Fe_3_O_4_). Facile synthesized nano-bio-composites were characterized by SEM, HR-TEM, XRD, FT-IR, XPS, TGA, and VSM analysis. Bare Fe_3_O_4_ nanoparticles (NPs), SCB-H, and SCB-H@Fe_3_O_4_ were tested for cytocompatibility and osteoconduction enhancement of human adipose tissue-derived mesenchymal stem cells (hADMSCs). The results confirmed the cytocompatible and nontoxic nature of SCB-H@Fe_3_O_4_. SCB-H did not show enhancement in osteoconduction, whilst on the other hand, Fe_3_O_4_ NPs exhibited a 0.5-fold increase in the osteoconduction of hADMSCs. However, SCB-H@Fe_3_O_4_ demonstrated an excellent enhancement in osteoconduction of a 3-fold increase over the control, and a 2.5-fold increase over the bare Fe_3_O_4_ NPs. Correspondingly, the expression patterns assessment of osteoconduction marker genes (ALP, OCN, and RUNX2) confirmed the osteoconductive enhancement by SCB-H@Fe_3_O_4_. In the proposed mechanism, the surface of SCB-H@Fe_3_O_4_ might provide a unique topology, and anchoring to receptors of hADMSCs leads to accelerated osteogenesis. In conclusion, agriculture waste-derived sustainable materials like “SCB-H@Fe_3_O_44_” can be potentially applied in highly valued medicinal applications of stem cell differentiation.

## 1. Introduction

Waste biomass exploitation for materials in tissue engineering applications is of huge importance and has not been fully exploited yet [1]. Sugarcane is the most cultivated crop in the world mainly for the food and alcohol industries [2]. Sugarcane bagasse (SCB) is available in abundance, and is mainly used for biofuel in electricity generation and as a substitute for pulp, paper, and board [3]. Structurally, SCB consists of cellulose (45–55%), hemicellulose (20–25%), and lignin (18–24%) [4]. Recently, SCB was found to be an impeccable source material for many applications [5,6,7]. However, its use as a scaffold in tissue engineering is still lacking. Previous tissue engineering and regeneration studies suggested that cellulose can be a candidate for scaffolds because of its low density and low toxicity with significant biodegradable properties [8]. Moreover, previous studies showed the biocompatibility of SCB-derived biogenic silica nanoparticles for biomedical applications [9]. Hydrothermal carbonization (HTC) is a thermal conversion process, known for converting biomass into carbonaceous solids, generally called hydrochar [10]. However, the carbonation of SCB and its functionalization as a scaffold material are of high interest and have not been exploited yet. Some other reusable materials such as quartz (silica microfibers) from the optical devices industry are used for scaffolds as introduced in the paper by G. Nechifor et al., 2019 [11]. Looking at the higher cost of extensively studied tissue engineering scaffold materials, carbonized SCB might provide a cheap source that can be functionalized and applied as a scaffold material. Hydrochar, formed during hydrothermal carbonization, has different functionalization patterns and chemical nanostructures [12]. Hydrochar is initially exploited frequently for soil amendments, but recent advancements in research and technology broadened its applications in energy production, carbon sequestration, gas adsorbents, agriculture, active carbon adsorbents, and bio-refinery [9]. This study approached the bio-medicinal application of SCB-derived hydrochar. Therefore, in this study, we proposed a new SCB hydrochar-Fe_3_O_4_ NPs-based scaffold that can be an innovative approach for bone regeneration scaffolds. As per the authors’ literature survey, this is the first report to apply SCB-based hydrochar modified with Fe_3_O_4_ NPs for the osteogenic enhancement of hADMSCs.

Bone defects mainly involve structural and functional defects or missing bone, caused by external factors, comminuted fractures, and neoplasm [13]. Stimulating the regeneration of injured bone via a biopolymer matrix with a stem cell is the recent research area under active investigations [14,15]. Stem cells are used for regeneration therapy because of their self-renewal ability and differentiation potency into multi-lineages, such as adipocyte, osteoblast, and neural cells [16]. Especially, human adipose tissue-derived mesenchymal stem cells (hADMSCs) have high chromosomal stability and more rapid proliferation with high cellular activity than other mesenchymal stem cells [17]. For these reasons, there is a lot of research on the therapeutic potential of the combination with hADMSCs, and the biomaterials are well-studied for bone regeneration [14]. In the bone tissue engineering field, recent research has been focused on the implantation of biomimetic scaffolds for osteoinduction and osteoconduction.

Iron oxide (Fe_3_O_4_) nanoparticles (NPs) were used to locate, track, and externally operate the nano-scaffolds and the target stem cells [18,19,20,21]. Besides, Fe_3_O_4_ NPs have shown osteogenic differentiation of mesenchymal stem cells [21,22]. Since Fe_3_O_4_ NPs are known to contribute to bone regeneration, “Fe_3_O_4_ NPs combined bone scaffolds materials” are better choices for bone healing [18]. There are different “Fe_3_O_4_ NPs combined scaffolds” including: 1. synthetic polymers (PLA [23], PLGA [24], PCL [25], and PEG [26]), 2. bio-macromolecules (collagen [27], silk fibroin [28], and chitosan [29]), 3. inorganic materials (bioactive glass [30] and hydroxyapatite [27]), 4. nano-supports (carbon nanotubes [31]), and 5. their complex of components [18]. However, cheaper, natural-origin, easily available, and biocompatible template scaffolds are highly important to access and enhance Fe_3_O_4_-based osteoconductive potentials.

In summary, this study aims for the hydrothermal carbonization of SCB to hydrochar (SCB-H), modification of SCB-H with Fe_3_O_4_ NPs (SCB-H@Fe_3_O_4_), and application of SCB-H@Fe_3_O_4_ for the osteoconduction enhancement of hADMSCs. This study also focuses on evaluating the gene expression patterns of osteogenic marker genes (ALP, OCN, and RUNX2) in the presence of the scaffold SCB-H@Fe_3_O_4_.

## 2. Experimental Methods

SCB was collected from Bhima Sahakari Sugar Factory (Takali-Sikandar, Solapur, Maharashtra, India). Sulfuric acid (H_2_SO_4_, GR grade) and iron (III) chloride hexahydrate (FeCl_3_·6H_2_O, GR grade) were obtained from DaeJung Chemical and Metals Co. Ltd. Gyeonggi-do, South Korea. Sodium sulfite (Na_2_SO_3_, ACS reagent grade) and sodium hydroxide (NaOH, ACS reagent grade) were received from Sigma Aldrich (St. Louis, MO, USA). SCB was washed with distilled water followed by drying in an oven at 90 °C for 24 h. The dried SCB was grounded in the grinder (BL811DKR, Tefal, Haute-Savoie, France) to obtain a very fine SCB powder. SCB powder (6 g) was taken into 200 mL of 2 M sulfuric acid solution. The solution was mixed thoroughly and then poured into the 250 mL Teflon Lined Hydrothermal Synthesis Autoclave Reactor (Beijing Getty Laboratory Glassware Co., Ltd. Beijing, China). The hydrothermal reactor was assembled carefully and kept at 180 °C for a 20 h reaction. The obtained black-colored SCB hydrochar (SCB-H) was separated from the solution and washed thoroughly with distilled water. The SCB-H was dried in an oven at 90 °C for 24 h. SCB-H modification with Fe_3_O_4_ NPs was done with the reduction–precipitation (R-P) method [32,33,34]. In a typical synthesis process, the SCB-H powder (1 g) was taken into 100 mL distilled water. The resultant mixture was ultra-sonicated by Sonics Vibra-Cell VC130 Ultrasonic Processor, power—130 W, frequency—20 kHz, and amplitude—60 µM (Sonics & Materials, Inc., Newtown, CT, USA) for 15 min. The well-dispersed SCB-H suspension was added with 100 mL of 3 % FeCl_3_.6H_2_O (GR grade) solution (prepared in 0.1 N HCl) and the mixture was mixed thoroughly for 15 min. Further, 60 mL of Na_2_SO_3_ solution (0.7 %) was added to this mixture. The addition of Na_2_SO_3_ solution turns the mixture color to red. Once the red color of the mixture turns back to the yellow, 1 M NaOH solution (60 mL) was added to the mixture. The developed black-colored precipitate of SCB-H modified with Fe_3_O_4_ (SCB-H@Fe_3_O_4_) was separated with an external magnet. The separated SCB-H@Fe_3_O_4_ was washed thoroughly with distilled water, ethanol, and methanol. The well-washed SCB-H@Fe_3_O_4_ was dried in an oven at 90 °C for 24 h. The dried SCB-H@Fe_3_O_4_ was powdered in a mortar and pestle and used in further experiments. For in vitro testing, all materials were ultra-sonicated by Sonics Vibra-Cell VC130 Ultrasonic Processor, power—130W, frequency—20 kHz, and amplitude—60 µM (Sonics & Materials, Inc., Newtown, CT, USA) for 10 min before every cell treatment.

The SCB-H and SCB-H@Fe_3_O_4_ surface morphology was observed by scanning electron microscopy (SEM, FC-SM10, Hitachi S-4800). The surface morphology and elemental mapping of the SCB-H@Fe_3_O_4_ were carried out by a high-resolution transmission electron microscope (HR-TEM, Tecnai G2 transmission electron microscope, Hillsboro, OR, USA). Functional group assignments of SCB-H and SCB-H@Fe_3_O_4_ were examined by Fourier transform infrared spectroscopy (FT-IR, Spectrum 100, PerkinElmer, Waltham, MA, USA). The crystalline purity of SCB-H and SCB-H@Fe_3_O_4_ was investigated by an X-ray powder diffractometer with Cu K_α_ radiation (λ = 1.5418 Å) (Ultima IV/Rigaku, Tokyo, Japan). Surface elemental analysis of SCB-H and SCB-H@Fe_3_O_4_ was done by X-ray photoelectron spectroscopy (XPS, Theta Probe AR-XPS System, Thermo Fisher Scientific, Dartford, UK). Thermogravimetric analysis (TGA) of SCB-H and SCB-H@Fe_3_O_4_ was performed from room temperature to 800 °C, at a heating rate of 10 °C/min, under N_2_ atmosphere using a thermal analyzer system (TA Instrument, Q600). Magnetic properties of Fe_3_O_4_ and SCB-H@Fe_3_O_4_ were analyzed by a vibrating sample magnetometer (VSM) (Lakeshore, Model: 7407, Carson, CA, USA).

The hADMSCs were purchased from Cell Engineering for Origin (CEFO), Seoul, Korea. The hADMSCs cells proliferated in the hADMSC growth medium (CEFO) supplemented with penicillin and streptomycin (Gibco, Gaithersburg, MD, USA). Cells were detached by using accutase (Millipore, Burlington, MA, USA) and seeded at a density of 2 × 10^4^ cells/cm^2^. To induce osteogenic differentiation, passage number 5 of cells was cultured in an osteogenic differentiation medium (OM) for 14 days. OM is composed of low-glucose DMEM (Gibco, Gaithersburg, MD, USA) with 1% penicillin-streptomycin (Gibco, Gaithersburg, MD, USA), 10% fetal bovine serum (Alphabioregen, Boston, MA, USA), 0.1 μM dexamethasone (Sigma Aldrich, St. Louis, MO, USA), 10 mM β-glycerophosphate (Sigma-Aldrich, St. Louis, MO, USA), and 50 μM L-ascorbic acid-2-phosphate (Sigma-Aldrich, St. Louis, MO, USA). Passage 4 of hADMSC was used for all experiments. All media were replaced every three days and all cells were cultured at 37 °C and 5% CO_2_. Cell viability was measured using Ez-cytox. For experiments, hADMSCs were seeded into 96-well plates (Nunclon, Waltham, MA, USA) and incubated with nano-scaffolds at various concentrations (10, 25, 50, 100, 200 µg/mL). To measure the short-term and long-term toxicity of nano-scaffolds, cells along with nano-scaffolds were cultured and differentiated for 24 h and 14 days, respectively. After incubation, the media with Ez-cytox reagent were treated into cells (DOGEN, Daejeon, Korea) and incubated for 2 h. Absorbance measurement at 450 nm was carried out by using a microplate reader (Molecular Devices, San Jose, CA, USA) and cell viability levels of nanoparticles were evaluated compared to non-treatment groups. Cell viability was calculated as a percentage of control mean viable cells.

After incubation with nano-scaffolds at concentrations 25, 50, and 100 µg/mL, osteogenic differentiation was evaluated by Alizarin Red S staining. Cells were rinsed with DPBS and fixed with 3.7% formalin (Sigma-Aldrich, St. Louis, MO, USA) for 15 min. After fixation, cells were stained with Alizarin Red S (Sigma-Aldrich, St. Louis, MO, USA) solution for 45 min at room temperature in the dark. To quantify Alizarin Red S staining, the dye was eluted with 10% cetylpiridium chloride (Sigma-Aldrich, St. Louis, MO, USA) and measured at an absorbance of 570 nm with an ELISA reader (Molecular Devices, San Jose, CA, USA). After incubation with nano-scaffolds at concentrations 25, 50, and 100 µg/mL, quantitative reverse transcription-polymerase chain reaction (qRT-PCR) analysis was carried out. Total RNA was extracted using Trizol reagent (Life Technologies, Karlsbad, CA, USA) and quantified using a NanoVue Spectrophotometer (GE Healthcare, Amersham, UK). cDNA was synthesized from 1 μg of total RNA using M-MLV Reverse Transcriptase (ELPISBIO, Gyeonggido, Korea) and used for qRT-PCR (CFX Connect™ Real-Time PCR Detection System; Bio-Rad, Hercules, CA, USA) with SYBR Green PCR Master Mix (KAPA, Wilmington, MA, USA). Amplification was performed using the following cycling program: initial denaturation at 95 °C for 3 min, 50 cycles of denaturation at 95 °C for 10 s, annealing at 60 °C for 10 s, and extension at 72 °C for 10 s. Immunofluorescence staining was done as follows: ADMSC cells were seeded on coverslips in a 6-well cell culture plate. After osteogenic differentiation, the cells were fixed with 3.7% formaldehyde for 15 min and treated with 0.25% Triton X-100 for 10 min. The cells were incubated with primary antibody and Alexa Fluor 555-conjugated secondary antibody (Cell Signaling Technology, Danvers, MA, USA) for 1 h each. The cellular nuclear was stained with 4′,6-diamidino 2-phenylindole (DAPI) (Sigma Aldrich, St. Louis, MO, USA). The fluorescence images were obtained by confocal microscopy (Olympus, Tokyo, Japan) and quantitative analysis was performed using ImageJ software (Version 1.41, Bethesda, MD, USA). Statistical analysis was done and all data are expressed as the mean ± SEM of three independent experiments. Statistical analysis was performed by one-way ANOVA with Tukey’s multiple comparison analysis. A *p*-value of less than 0.05 was considered statistically significant.

## 3. Results and Discussion

**Scheme 1:**
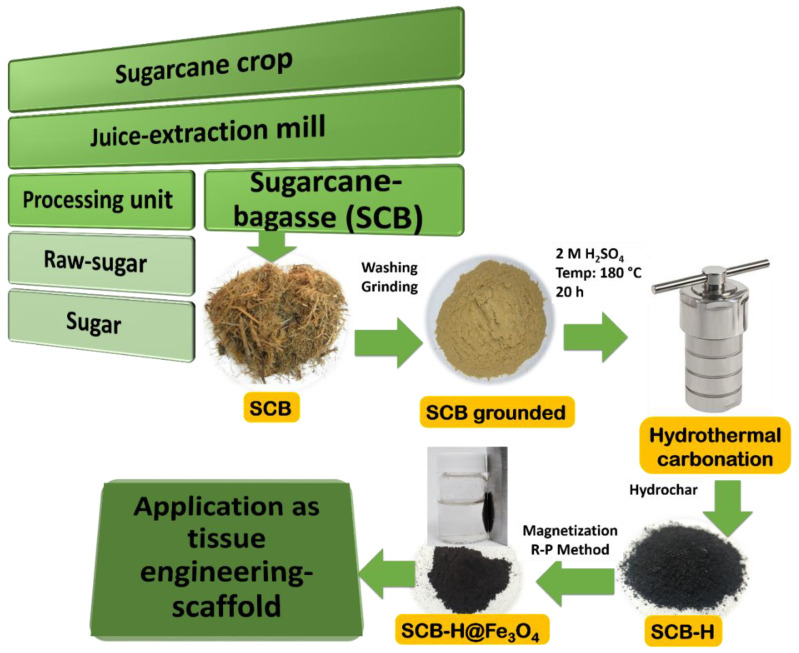
Flow chart of sugarcane bagasse (SCB) source and synthesis process of SCB-H@Fe_3_O_4_.

Internationally, it was projected that around 540 million tons per year of SCB is produced [35]. In the sugar mill, the sugarcane collected from agriculture is squashed for juice extraction and the obtained fibrous residual substance of sugarcane is termed as bagasse [36]. Scheme 1 describes the flow of obtaining SCB from agricultural sources. Among all available biomasses, SCB has some unique advantages such as huge production, industrial availability, and its cleanliness (as sugarcane undergoes repeated extraction and water soaking cycles in the sugar mill) [37]. It is very important to break the tight lingo-cellulosic SCB structure before its utilization as a valuable material [37]. In this study, we hydrolyzed the SCB lignocellulosic structure by acid-based hydrothermal carbonation experiments. SCB was successfully carbonized to form SCB-H by the hydrothermal method in Scheme 1. In this hydrothermal process, biomass SCB was transformed into a black-colored carbon mass, mainly by hydrolysis and dehydration processes under the harsh conditions of acid (H_2_SO_4_), high temperature, and high pressure [38]. The SCB-H was further magnetized (Fe_3_O_4_ NPs) by the reduction–precipitation (R-P) method to form SCB-H@Fe_3_O_4_ (Scheme 1). In the detailed process of the R-P method, after the attachment of the Fe^3+^ ions to the SCB-H surface, the reductant Na_2_SO_3_ was added. The Na_2_SO_3_ caused the partial reduction of these Fe^3+^ to Fe^2+^ ions. Further, the addition of precipitant NaOH leads to the formation of Fe_3_O_4_ NPs on the SCB-H surface. This R-P method is inexpensive, free from reactive gases and surfactants, and rapid for the synthesis of Fe_3_O_4_ NPs [32]. The facile synthesized SCB-H@Fe_3_O_4_ was washed thoroughly several times with water, ethanol, and methanol, and was finally autoclaved, ultrasonicated, and used for the osteogenic application.

**Figure 1:**
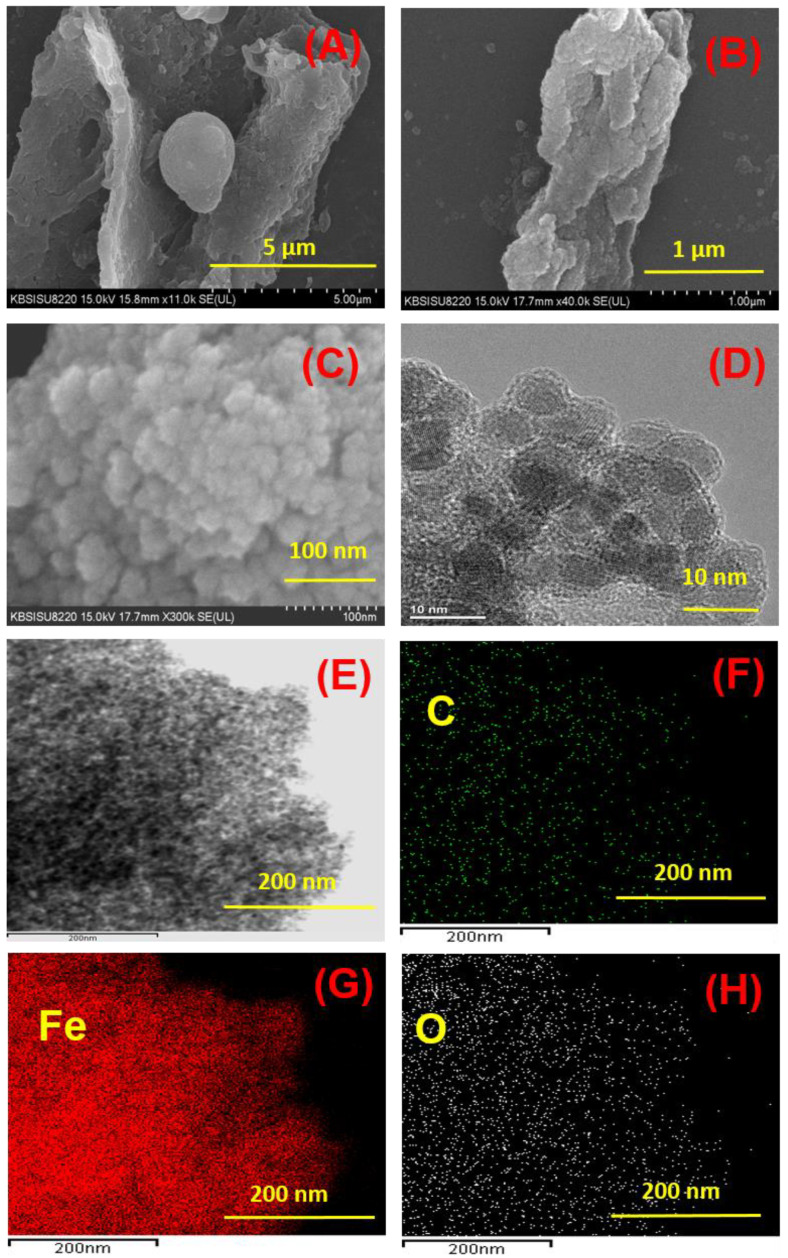
SEM image of (**A**) SCB hydrochar (SCB-H) and (**B**) SCB-H@Fe_3_O_4_, (**C**) the zoomed (100 nm) surface of SCB-H@Fe_3_O_4_, (**D**) HR-TEM image of the SCB-H@Fe_3_O_4_ surface, (**E**) HAADF-STEM image of SCB-H@Fe_3_O_4_, and EDS elemental mapping images of (**F**) carbon (C), (**G**) iron (Fe) and (**H**) oxygen (O).

The detailed morphological structures were observed by SEM and HR-TEM analysis and the structural characterizations were completed by XRD, FT-IR, XPS, TGA, and VSM analysis. Figure 1 shows the SEM images of SCB-H and SCB-H@Fe_3_O_4_ and a zoomed view of the SCB-H@Fe_3_O_4_ surface. The surface of SCB was smooth and undistorted [39]. In SCB-H, the hydrolysis of the strong lignocellulosic structure of SCB during the hydrothermal process was evident. Hydrothermal treatment under hostile conditions of acid (H_2_SO_4_), high temperature, and high pressure caused the hydrolysis and dehydration of the SCB surface and led to the distorted morphology of SCB-H (Figure 1A). In the hydrothermal process, the SCB structure was broken and led to the formation of different shapes of hydrochar particles. The oval-shaped structure of hydrochar particles of SCB is shown in Figure 1A. However, the surface of SCB-H@Fe_3_O_4_ (Figure 1B) shows the changed surface morphologies. However, the 1 µm-magnification scale Fe_3_O_4_ NPs were not visible on the SCB-H@Fe_3_O_4_ surface (Figure 1C). Therefore, to observe the morphologies of Fe_3_O_4_ NPs over the SCB-H@Fe_3_O_4_ surface, the zoomed view (100 nm) of the SCB-H@Fe_3_O_4_ surface was taken (Figure 1C). It evidenced a loading of the small circular-shaped nanoparticulate structure of Fe_3_O_4_ NPs throughout the SCB-H surface (Figure 1C). Therefore, the SEM analysis confirmed the distortion of the SCB structure in SCB-H and distorted SCB-H, provided the additional attachment sites for Fe_3_O_4_ loading.

Furthermore, the closer surface inspection of SCB-H@Fe_3_O_4_ was made by HR-TEM image analysis (Figure 1D). The obtained image displayed the SCB-H surface modification with Fe_3_O_4_ nanoparticles (Figure 1D). The observed Fe_3_O_4_ nanoparticles had a circular shape with a 5–8 nm diameter (Figure 1D).

Moreover, the surface elemental mapping was performed using the high-angle annular dark-field scanning transmission electron microscopy with energy-dispersive HAADF-STEM and energy-dispersive X-ray spectroscopy (EDS) (Figure 1E). Figure 1F–H display the TEM-EDS elemental mapping of SCB-H@Fe_3_O_4_. The obtained distribution of C, Fe, and O designates the significant SCB-H surface modifications with Fe_3_O_4_ (Figure 1F–H). Thus, the entire SEM and TEM analyses confirm the fine-tuning of the SCB-H surface with Fe_3_O_4_ NPs.

**Figure 2:**
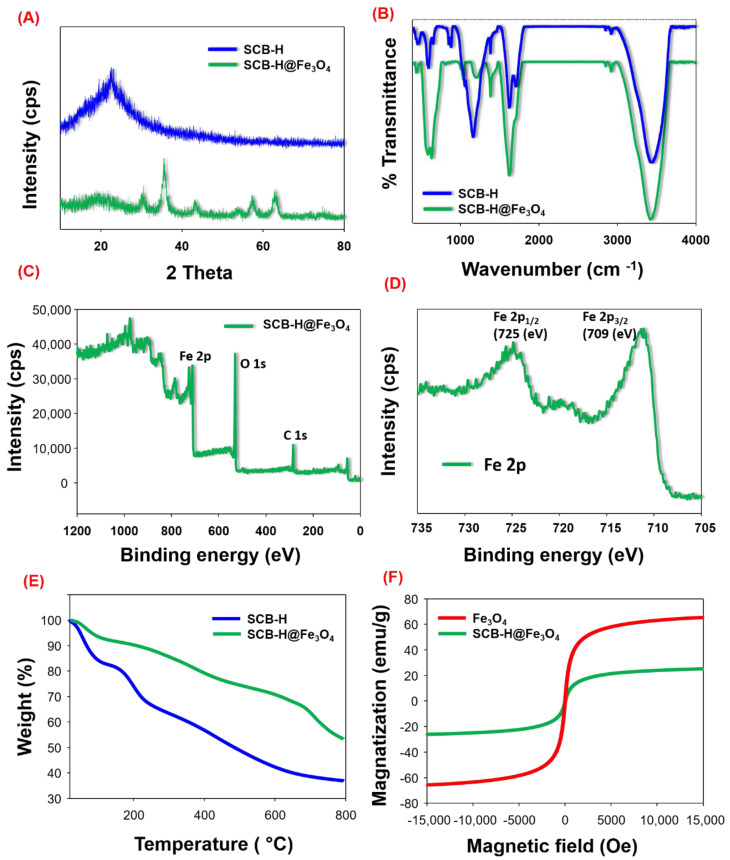
XRD (**A**) and FT-IR (**B**) analysis of SCB-H and SCB-H@Fe_3_O_4_, (**C**) XPS analysis of SCB-H@Fe_3_O_4_, (**D**) high-resolution spectrum of Fe 2p**,** TGA analysis of SCB-H and SCB-H@Fe_3_O_4_ (**E**), and VSM analysis of Fe_3_O_4_ and SCB-H@Fe_3_O_4_ (**F**).

The crystalline nature of SCB-H and SCB-H@Fe_3_O_4_ was characterized by XRD analysis. Figure 2A shows the XRD patterns of SCB-H and SCB-H@ Fe_3_O_4_. The XRD pattern of SCB-H exhibited a strong diffraction peak (002) at about a 2θ value of 22° (Figure 2A). The obtained diffraction peak (002) corresponds to the structure of various amorphous layers [40]. However, the XRD pattern of SCB-H@ Fe_3_O_4_ displays the diffraction peaks at 2θ values of 22.02°, 30.46°, 35.80°, 43.37°, 53.88°, 57.14°, and 63.37° (Figure 2A). The diffraction peak at 22.02° corresponds to the (002) crystalline plane of SCB-H. Moreover, the diffraction peaks at 2θ values of 30.46°, 35.80°, 43.37°, 53.88°, 57.14°, and 63.37° correspond to the crystalline planes (220), (311), (400), (422), (511), and (440), respectively, of the pure cubic spinal structure of Fe_3_O_4_ [34]. Hence, the XRD patterns strongly confirm the successful synthesis of SCB-H@ Fe_3_O_4_.

Further, the FT-IR analysis was carried out to assign the functional groups from samples (Figure 2B). The FT-IR analysis of SCB-H and SCB-H@Fe_3_O_4_ samples showed the common absorption bands of 3427, 2925, 2841, 1626, 1386, and 1035 cm^−1^ corresponding to the O-H stretching vibrations in -COOH or -OH groups, aromatic-aliphatic stretching in (C-H), aromatic-aliphatic stretching in (C-H) modes of benzene, C=C vibrations of aromatic rings, C-O stretching vibrations in ester, hydroxyl, or ether, and C-OH stretching of primary alcohols, respectively. A similar FT-IR peaks assignment pattern of SCB-hydrochar was reported by Buapeth et al., 2019 [41]. However, the strong band at 1724 cm^−1^ corresponding to (C=O) vibrations of carboxyl groups was present in SCB-H and absent in SCB-H@Fe_3_O_4_. This might be due to the interaction of the Fe_3_O_4_ NPs with the carboxyl groups of SCB-H. In the stepwise mechanism of Fe_3_O_4_ loading on SCB-H, firstly, the Fe^3+^ ions bind to the free hydroxyl/carboxyl groups present on the SCB-H surface. Then, after the addition of Na_2_SO_3_, Fe^3+^ ions are partially reduced to Fe^2+^. In the next step of NaOH addition, Fe^3+^ and Fe^2+^ ions get precipitated and form the Fe_3_O_4_ nanoparticles on the surface of SCB-H. Importantly, SCB-H@Fe_3_O_4_ was displayed at 646 and 574 cm^−1^, which are very strong absorption bands corresponding to the metal oxide Fe-O bond [42,43]. These acquired FT-IR results highlighted a successful SCB-H@Fe_3_O_4_ synthesis.

Figure 2C shows the XPS analysis of SCB-H@Fe_3_O_4_. The XPS survey spectrum of SCB-H@Fe_3_O_4_ showed binding energies of 284.6, 532.18, and 711.14 eV, corresponding to the C 1s, O 1s, and Fe 2p elements, respectively (Table S1, Supplementary Information). The presence of Fe 2p in SCB-H@Fe_3_O_4_ evidenced the Fe_3_O_4_ modification of SCB-H. Moreover, the high-resolution XPS spectrum of the Fe 2p peak showed typical Fe 2p_1/2_ (725 eV) and Fe 2p_3/2_ (709 eV) peaks that are characteristics for pure Fe_3_O_4_ NPs (Figure 2D). The atomic concentration of elements C 1s, Fe 2p, and O 1s was found to be 33.81, 13.86, and 50.80%, respectively (Table S1, Supplementary Information). The obtained XPS results are in strong agreement with the XRD and FT-IR characterizations and hence proved the successful synthesis of SCB-H@Fe_3_O_4_.

Thermal characteristics of SCB-H and SCB-H@ Fe_3_O_4_ were carried out by TGA analysis (Figure 2E). The details of all values in the TGA analysis were mentioned in Table S2, Supplementary Information. The TGA curve of SCB-H shows the typical four stages of thermal degradation: 30–140, 140–353, 353–500, and 500–800 °C. The first stage of 30–140 °C gave a weight loss of 17.43% corresponding to the loss of moisture. The degradation stages of 140–353, 353–500, and 500–800 °C correspond to the breakdown of the hemicellulose, cellulose, and lignin structure [44]. However, the SCB-H@Fe_3_O_4_ sample showed completely different thermal characteristics (Table S2, Supplementary Information). Thus, it sustains the successful modification of SCB-H. SCB-H@Fe_3_O_4_ exhibited five stages of thermal degradation: 30–187, 187–302, 302–567, 567–654, and 654–800 °C (Figure 2E). The initial weight loss of 8.524 % at 30–187 °C was observed at the first stage due to the moisture loss from the material. Further stages of thermal decomposition corroborated the degradation of the hemicellulose, cellulose, and lignin structure. The total residue that remained after the thermal degradation process after 800 °C was 36.96% for SCB-H, and 53.48% for SCB-H@Fe_3_O_4_ (Figure 2E). Therefore, the Fe_3_O_4_ modification of SCB-H enhanced the thermal stability of SCB-H@Fe_3_O_4_. All thermal analyses confirmed the changed thermal degradation patterns and successful synthesis of SCB-H@Fe_3_O_4_.

The magnetism of Fe_3_O_4_ combined tissue engineering scaffolds plays a crucial role in the tracing, locating, and mapping of the progress of the response tissue [19,24,27]. Therefore, it was a prerequisite to know the magnetic potential of the SCB-H@Fe_3_O_4_ and bare Fe_3_O_4_ NPs. To investigate the magnetic characteristics, VSM analysis was carried out (Figure 2F). The VSM analysis showed a very high magnetization potential (magnetic saturation values) of SCB-H@Fe_3_O_4_ and bare Fe_3_O_4_ NPs at 25.38 and 65.66 emu/g, respectively. The magnetic potential of bare Fe_3_O_4_ NPs was higher compared to SCB-H@Fe_3_O_4_. These results confirmed the successful impregnation of Fe_3_O_4_ NPs on SCB-H.

The impregnation of Fe_3_O_4_ NPs with SCB-H was caused by an increase in mass and hence led to a decrease in the magnetization of SCB-H@Fe_3_O_4_ compare to the bare Fe_3_O_4_ NPs (Figure 2F). Similar results of decreasing magnetization after loading of laccase on modified halloysite nanotubes was observed before by [45,46]. Scheme 1 shows the visual view of separated SCB-H@Fe_3_O_4_ from water by the magnet. Both the samples showed a typical magnetic hysteresis curve, where the coercivity and remanence values were zero. Thus, this confirmed the superparamagnetic nature of SCB-H@Fe_3_O_4_.

The hydrochar, produced from organic materials by hydrothermal carbonization, is a carbon-based material. The organic material undergoes dehydration and carbonization during hydrothermal carbonization and transforms into colloidal carbon particles [47]. The molecular structure of hydrochar has been proposed in various models. The structure models reported in the literature have a common feature that the aromatic ring consists of carbon and oxygen [47,48,49]. These various molecular models allow hydrochar to be synthesized and utilized for specific applications [12]. Therefore, it is a prerequisite to evaluate the cytotoxic studies of SCB-H and SCB-H@Fe_3_O_4_.

**Figure 3:**
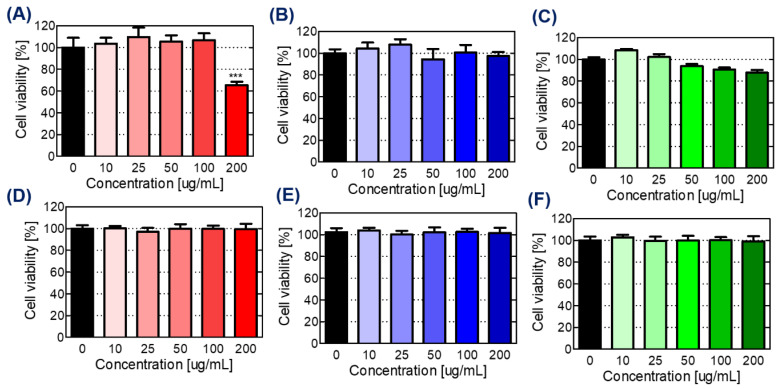
Cytotoxicity of studied materials against the hADMSCs. (**A**–**C**) hADMSCs viability measured after 24 h, and (**D**–**F**) hADMSCs viability measured after 14 d. Nano-scaffolds; (**A**,**D**) Fe_3_O_4_ (**B**,**E**) SCB-H, and (**C**,**F**) SCB-H@Fe_3_O_4_. *** *p* < 0.001.

The detailed results regarding the cytotoxicity evaluation are shown in Figure 3. To evaluate the SCB-H@Fe_3_O_4_ toxicity to hADMSCs cells, both long-term (14 d) and short-term (24 h) toxicity analyses were made at varying concentrations of 10 to 200 µg/mL. The obtained results revealed that Fe_3_O_4,_ SCB-H and SCB-H@Fe_3_O_4_ exhibit strong cytocompatibility until the 100 µg/mL concentration, and a very low level of cytotoxicity was observed at 200 µg/mL after 24 h of incubation (Figure 3). However, all the nanocomposites did not show a cytotoxicity effect after the 14-d incubation. This is mainly due to the recovery of the damaged cells after the 14-d incubation. Mostly, the size of a particle smaller than 100 nm is found to be toxic to humans [50,51]. However, the material used in this study as a scaffold is more than 1μm in size, therefore it is less toxic to humans. Therefore, the obtained results confirmed that the materials are cytocompatible, and the 100 µg/mL concentration can be used for further osteoconduction enhancement studies. Hence, once cytocompatibility results were confirmed, the osteoconduction experiments were carried out.

**Figure 4:**
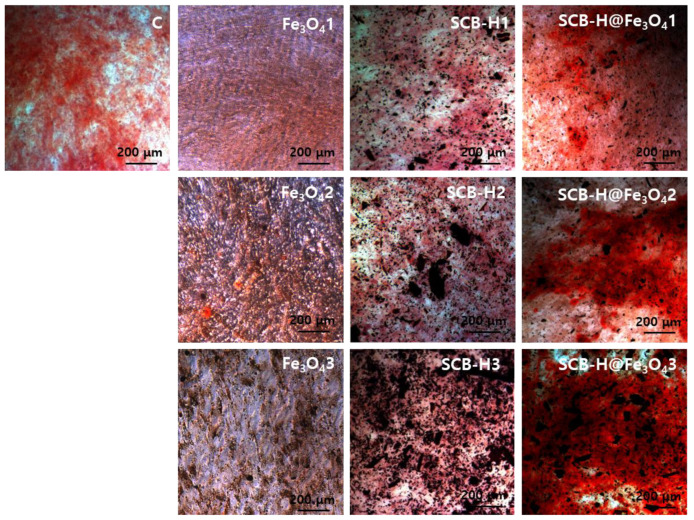
Analysis of calcium deposition by ARS staining. hADMSCs were differentiated to osteoblasts for 14 days in osteogenic induction media with or without nano-scaffolds. After differentiation, the calcium deposition was determined with ARS staining. Intracellular and extracellular calcium was stained to red by ARS. C: vehicle, 1: 25 μg /mL, 2: 50 μg /mL 3: 100 μg/mL.

**Figure 5:**
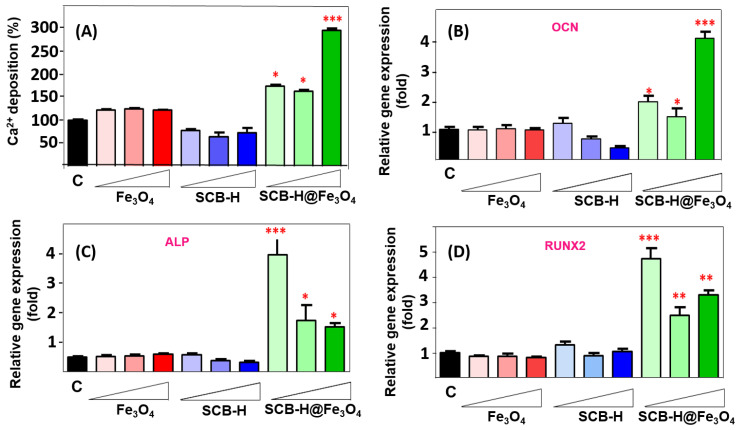
Quantification of osteogenesis. hADMSCs were differentiated to osteoblasts for 14 days in osteogenic induction media with or without nano-scaffolds. After differentiation, the osteogenic markers were quantified by using ARS staining and real-time RT PCR. (**A**) Quantification of ARS staining. (**B**–**D**) mRNA level of osteogenic marker. C: vehicle, nano-scaffolds concentration from 25, 50 to 100 µg/mL for every sample of bare Fe_3_O_4_, SCB-H, and SCB-H@Fe_3_O_4_. * *p* < 0.05, ** *p* < 0.01 *** *p* < 0.001 in comparison with untreated cells.

The waste-to-wealth concept goals encourage a future sustainable lifestyle, where waste valorization is not only just seen as its fundamental benefits to the environment but also to improve new advanced technologies and better livelihoods [7,52]. This study investigates the osteoconduction effect of novel Fe_3_O_4_ functionalized SCB-based hydrochar materials. The osteoconduction of bare Fe_3_O_4_, SCB-H, and SCB-H@Fe_3_O_4_ on hADMSCs was investigated at the concentration of the 25, 50, and 100 µg/mL. In the typical osteogenic differentiation process, first, the osteoblasts are formed. Further, these osteoblasts form bone nodules with extracellular calcium depositions. Intracellular and ECM calcium deposition indicates the maturation of osteoblasts differentiation, especially at the late stage of osteogenesis [20,53]. This osteogenic differentiation of hADMSCs was evaluated by ARS staining. The ARS staining for investigating the calcium deposition is shown in Figure 4. The ARS staining images display the enhanced calcium deposits in SCB-H@Fe_3_O_4_ compared to the bare Fe_3_O_4_ and SCB-H samples (Figure 4). An increase in calcium deposition was observed with a subsequent increase in the concentrations of SCB-H@Fe_3_O_4_ (25 to 100 µg/mL) (Figure 4). Furthermore, the quantitative determination of calcium deposition is shown in Figure 5A. The obtained results are in agreement with Figure 4.

The small increase in the calcium deposit was noted in the bare Fe_3_O_4_ NPs (Figure 5A). This observation is supported by a previous report [17]. Conversely, SCB-H did not display the osteoconduction enhancement of hADMSCs (Figure 5A). However, the nano-bio-composite SCB-H@Fe_3_O_4_ gave significantly enhanced calcium deposits than bare Fe_3_O_4_ and SCB-H samples. The SCB-H@Fe_3_O_4_ exhibited a 3-fold increase in the calcium deposits compared to the control and a 2.5-fold increase compared to the Fe_3_O_4_ NPs (Figure 5A). Thus, the accelerated osteoconduction of hADMSCs was observed with SCB-H@Fe_3_O_4_. Since the Fe_3_O_4_ NPs are known to possess osteoconduction potential, Fe_3_O_4_ NPs combined bone scaffolds materials are the better choices for bone healing and enhancing the osteoconduction potential of bare Fe_3_O_4_ NPs [18]. It was reported that the surface of particles affects the distribution and deformation of the cell membrane, including the adhesion protein responsible for cell–cell interactions [54]. Further, nano-micro-scaffold patterns modulate stem cell differentiation and different stem cell lineages by regulating cell extension and affinity [55]. Especially, the cell surface of the particle is very crucial for hADMSCs which depend on the cell adhesion signal. It is well-known that the dynamic differentiation of hADMSCs is induced by the condition of the microenvironment [56]. In the case of Fe_3_O_4_, the properties of small size and charge may interfere with cell activity. It was reported that nanoparticles can block the receptor [57]. A small amount of the osteoconduction obtained in Fe_3_O_4_ NPs might be due to the aggregation effect. Further, SCB-H is a porous structure that might affect cell attachment and migration in vitro, and mean pore size is known to influence cell phenotypic expression and its morphology [58]. It seems that SCB-H serves as a template for stabilizing the Fe_3_O_4_ NPs in its porous structures and significantly enhances the osteogenesis process in hADMSCs. The small size of Fe_3_O_4_ NPs fits in the pores of SCB-H and gets stabilized, and this might provide the optimal microenvironment and multipoint attachments to hADMSC which leads to enhanced osteogenesis. Therefore, keeping a view on all these points, we proposed the mechanism that SCB-H provides a suitable surface to bind Fe_3_O_4_ NPs, and the SCB-H-bound Fe_3_O_4_ NPs provide unique epitopes and multipoint attachments to the hADMSCs and affect the cell microenvironment, which leads to the accelerated osteoconduction of hADMSCs. Since SCB-H is derived from agricultural waste biomass with a very simple synthesis process, it is several folds more economical than the carbon-based structures, such as graphene and carbon nanotube. The graphene and carbon nanotubes have been extensively studied for bone regeneration applications [59,60]. However, this study might open a new window to look at very easily available biomass materials, convert them into functionalized nano-biomaterials, and finally apply them in the recent medicinal applications such as stem cell regeneration. As per the authors’ literature survey, there is no such report for the use of hydrochar-based materials as a support for either osteoconductive or bore regeneration materials.

**Figure 6:**
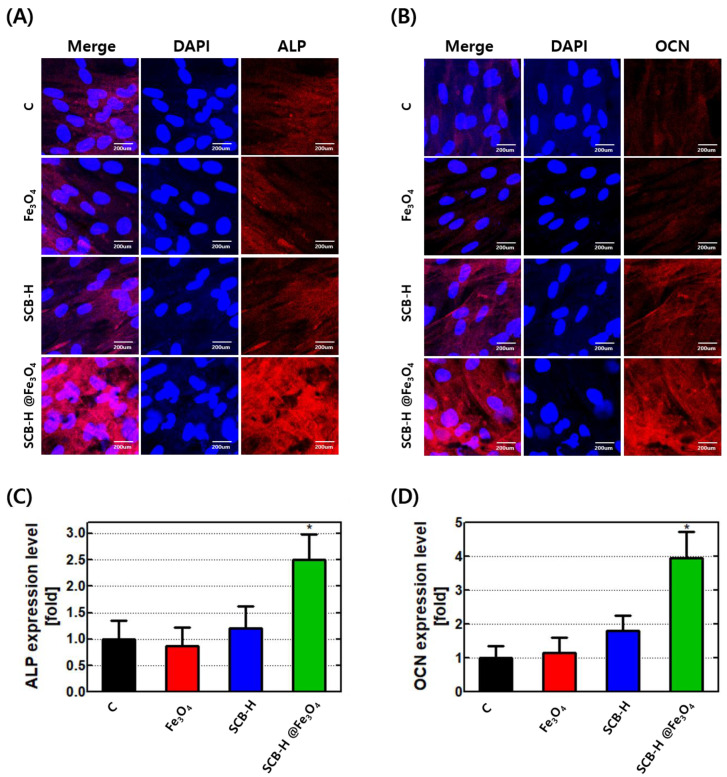
The protein levels of OCN and ALP. hADMSCs were differentiated to osteoblasts for 14 days in osteogenic induction media with or without nano-scaffolds. After differentiation, the protein levels of osteogenic markers (**A**) ALP and (**B**) OCN were analyzed by immunocytochemistry. All results were quantified using ImageJ Software (**C**) ALP and (**D**) OCN. All materials are treated with 100 µg/mL for every cell. C: vehicle, * *p* < 0.05.

After confirming the osteogenesis enhancement (Figure 4 and Figure 5A), it is very important to know the expression patterns of the osteogenic marker genes. There are mainly three osteogenic markers such as osteocalcin (OCN), alkaline phosphatase (ALP), and runt-related transcription factor 2 (RUNX2), that indicate the early and later stages of osteogenesis, respectively. To evaluate the early stage of osteogenesis, ALP and RUNX2 are used. However, OCN is used for the later stage of osteogenesis. The expression patterns of OCN, ALP, and RUNX2 from cells incubated with bare Fe_3_O_4_, SCB-H, and SCB-H@Fe_3_O_4_ (at concentrations of 25, 50, and 100 µg/mL) are presented in Figure 5B–D. The results revealed that when hADMSCs cells are incubated with SCB-H@Fe_3_O_4_, the OCN, ALP, and RUNX2 genes have a 7-fold higher expression than the control, bare Fe_3_O_4_, and SCB-H (Figure 5B–D). However, when hADMSCs cells are incubated with bare Fe_3_O_4_ and SCB-H, the OCN, ALP, and RUNX2 genes do not show an increased level of gene expression (Figure 5B–D). The protein levels of OCN and ALP are also consistent with the gene expression level (Figure 6A–D). The cells with SCB-H@Fe_3_O_4_ have more expression levels of ALP and OCN, while SCB-H and Fe_3_O_4_ do not have a significant change (Figure 6A–D). As a result, the obtained osteogenic marker gene expression patterns strongly corroborated the acceleration of osteoconduction by SCB-H@Fe_3_O_4_.

Moreover, when we closely observe the expression patterns of SCB-H@Fe_3_O_4_, the obtained results indicate that the gene expression level of OCN is significantly increased in the “SCB-H@Fe_3_O_4_-treated cells” in a dose-dependent manner (Figure 5B). It was consistent with calcium deposition results (Figure 5A). The OCN was expressed higher (4-fold) at the 100 µg/mL concentration of SCB-H@Fe_3_O_4_ than the control. On the other hand, the gene expression level of ALP and RUNX2 at the higher concentration (100 µg/mL) of SCB-H@Fe_3_O_4_ was significantly less than the lower concentration (25 µg/mL) of SCB-H@Fe_3_O_4_. To understand these marker genes dynamics defending upon the SCB-H@Fe_3_O_4_ concentrations, it is very important to know the markers (OCN, ALP, and RUNX2) in detail. ALP is mainly expressed in the early stage of osteogenesis, and initial osteogenesis events and ALP expression are mainly regulated by RUNX2 [61]. RUNX2 is best known as a master regulator in osteoclast development and function. RUNX2, one of the transcription factors, is essential for the commitment of mesenchymal stem cells to the osteoblast lineage. The expression level of RUNX2 increases in the early stage of osteogenesis. Once the mature osteoblasts form, in the later stage of osteogenesis, the RUNX2 expression decreases. Therefore, the regulation of gene expression by RUNX2 is a positive manner at the early stage of osteogenesis, while RUNX2 inhibits the process at the later stage [62]. Conversely, OCN, a metabolic regulator of the bone microenvironment, is used as a marker for the later stage of osteogenesis (for mature osteoblasts) or bone formation because OCN is secreted by only by mature osteoblast [63].

Accordingly, in Figure 5B–D, the dominant expression of ALP and RUNX2 markers at lower concentrations of SCB-H@Fe_3_O_4_ indicates the early stage of the osteogenesis, however, dominant OCN at higher concentrations (100 µg/mL) of SCB-H@Fe_3_O_4_ indicates the later stage of osteogenesis (mature osteoblast or bone formation). These obtained results are in strong agreement with the 3-fold increase in calcium deposition of 100 µg/mL SCB-H@Fe_3_O_4_ (Figure 5A). The overall osteogenic marker gene expression results corroborated the accelerated osteogenesis by SCB-H@Fe_3_O_4_, and also confirmed the SCB-H@Fe_3_O_4_ concentration-defendant osteogenesis of hADMSCs. Therefore, all the necessary parameters, “calcium staining/quantification, osteogenic marker gene expression patterns, and finally immunochemistry evaluations”, established the osteoconductive tissue engineering potential of waste biomass-derived SCB-H@Fe_3_O_4_.

## 4. Conclusions

This study explores the valorization of discarded waste biomass SCB to modified SCB-H@Fe_3_O_4_ as value-added nano-biomaterials applicable in stem cell differentiation. Its sustainable origin makes this nano-biomaterial contribute to the growth of a “circular, green, and bio-economy”. SCB-H@Fe_3_O_4_ was successfully synthesized and characterized. It efficaciously accelerated the osteogenesis of hADMSCs. The detailed evaluations confirmed the significant potential of SCB-H@Fe_3_O_4_ in osteoconduction enhancement. The osteogenic marker evaluations revealed the concentration-dependent osteogenesis stages. Additionally, the magnetic property of SCB-H@Fe_3_O_4_ can offer imaging of the target tissue. This study assures that agricultural waste materials can be hydrothermally carbonized and magnetically functionalized for highly valued medicinal applications. The enormous availability of waste biomass, cheap cost of carbonization, and easy functionalization can give important nano-biomaterials like SCB-H@Fe_3_O_4_ application like in the osteogenic differentiation of hADMSCs.

## Supplementary Materials

The following are available online at https://www.mdpi.com/2079-4991/10/9/1793/s1, Table S1: Element, binding energies, and atomic concentrations (%) from XPS analysis of SCBH@Fe_3_O_4_., Table S2: The detailed thermal characteristics of SCB-H and SCB-H@ Fe_3_O_4_.
