# Supplementary material for: Supermagnetic Sugarcane Bagasse Hydrochar for Enhanced Osteoconduction in Human Adipose Tissue-Derived Mesenchymal Stem Cells
Source: Nanomaterials (Basel). 2020 Sep 9;10(9):1793. doi: 10.3390/nano10091793 (PMC7557583; doi:10.3390/nano10091793)
Supplement: Supplementary file 1 [file nanomaterials-10-01793-s001.pdf]

nanomaterials

Supplementary Materials

Supermagnetic Sugarcane Bagasse Hydrochar for Enhanced Osteoconduction in Human Adipose Tissue-Derived Mesenchymal Stem Cells

Min Kim [1], Seung-Cheol Jee [1], Jung-Suk Sung [1] and Avinash A. Kadam [2,*]

[1] Department of Life Science, College of Life Science and Biotechnology, Dongguk University-Seoul, 32, Dongguk-ro, Ilsandong-gu, Goyang-si, Gyonggido 10326, Korea; pipikimmin@naver.com (M.K.); markjee@naver.com (S.-C.J.); sungjs@dongguk.edu (J.-S.S.)

[2] Research Institute of Biotechnology & Medical Converged Science, Dongguk University-Seoul, 32, Dongguk-ro, Ilsandong-gu, Goyang-si, Gyonggido 10326, Korea

* Correspondence: avikadam2010@gmail.com or kadamavinash@dongguk.edu; Tel.: +82-31-961-5616; Fax: 82-31-961-5108

Table S1. Element, binding energies and atomic concentrations (%) from XPS analysis of SCBH@Fe_3O_4.

Element Name	Binding Energy (eV)	Atomic% Concentration
C 1s	284.6	33.81
Fe 2p	711.14	13.86
O 1s	532.18	50.80

Table S2. The detailed thermal characteristics of SCB-H and SCB-H@ Fe_3O_4.

Material Studied	TGA		
	**Stages of Thermal Degradation (°C)**	**Weight Loss (%) in the Stage**	**Amount Loss in (mg) in the Stage**
SCB-H	30–140	17.43	0.832
	140–353	19.41	0.927
	353–500	13.04	0.623
	500–800	13.17	0.622
SCB-H@Fe_3O_4	30–187	8.524	0.750
	187–302	5.921	0.521
	302–567	12.15	1.070
	567–654	5.77	0.508
	654–800	14.14	1.25

© 2020 by the authors. Licensee MDPI, Basel, Switzerland. This article is an open access article distributed under the terms and conditions of the Creative Commons Attribution (CC BY) license (http://creativecommons.org/licenses/by/4.0/).
